# Compositions and Structural Geometries of Scaffolds Used in the Regeneration of Cleft Palates: A Review of the Literature

**DOI:** 10.3390/polym14030547

**Published:** 2022-01-28

**Authors:** Víctor A. Reyna-Urrutia, Arely M. González-González, Raúl Rosales-Ibáñez

**Affiliations:** Laboratorio de Ingeniería Tisular y Medicina Traslacional, Facultad de Estudios Superiores Iztacala, Universidad Nacional Autónoma de México, Tenayuca-Chalmita S/N, Cuautepec Barrio Bajo, Alcaldía Gustavo A. Madero, Ciudad de México CP 07239, Mexico; victor_r03_urrutiaa@hotmail.com (V.A.R.-U.); arely.gonzalez@iztacala.unam.mx (A.M.G.-G.)

**Keywords:** cleft palate, three-dimensional scaffolding, tissue engineering, manufacturing technique

## Abstract

Cleft palate (CP) is one of the most common birth defects, presenting a multitude of negative impacts on the health of the patient. It also leads to increased mortality at all stages of life, economic costs and psychosocial effects. The embryological development of CP has been outlined thanks to the advances made in recent years due to biomolecular successions. The etiology is broad and combines certain environmental and genetic factors. Currently, all surgical interventions work off the principle of restoring the area of the fissure and aesthetics of the patient, making use of bone substitutes. These can involve biological products, such as a demineralized bone matrix, as well as natural–synthetic polymers, and can be supplemented with nutrients or growth factors. For this reason, the following review analyzes different biomaterials in which nutrients or biomolecules have been added to improve the bioactive properties of the tissue construct to regenerate new bone, taking into account the greatest limitations of this approach, which are its use for bone substitutes for large areas exclusively and the lack of vascularity. Bone tissue engineering is a promising field, since it favors the development of porous synthetic substitutes with the ability to promote rapid and extensive vascularization within their structures for the regeneration of the CP area.

## 1. Introduction

The growth and development disorders associated with the craniofacial region are varied; within these, nasolabial and nasopalatine clefts are considered the most frequent, also known as cleft lip (CL) and cleft palate (CP), respectively. Patients with cleft palates are more likely to have deficiencies in both speech and language. Similarly, there have been statistics showing that children with cleft lip or cleft palate have higher rates of learning problems than the general population and also show abnormalities in brain morphology [1].

Embryologically, the nasopalatine cleft is constituted by the horizontal processes typical of the maxillary process, which form around the seventh week of gestation. The borders of mesenchymal tissues, which are covered by epithelial tissues, must migrate towards the midline and its posterior union with the contralateral portion, giving rise to the floor of the nostrils and roof or palatine vault. The above develop from the apoptosis phenomenon, whereby the epithelial cells of the coating of said medial edges of both processes are expressed, allowing the exposure of the mesenchymal tissue; the subsequent migration, contact and union of these processes; and finally their re-epithelialization. Apparently, in the case of CP, this apoptotic process does not develop at the epithelial border of the palatal processes, and is sometimes aided by the presence of the tongue in the fissure, which under normal conditions should descend to allow this union [2,3].The use of autogenous bone grafts is considered the main option for PC bone regeneration, since it minimizes the consequences of storage; in addition, the reconstruction is carried out at the same time as the extraction of the bone that is used to repair the affected area [4]. Another approach that can be taken to regenerate the PC area is the use of allogeneic bone, although because there is a risk of disease transmission, this is only considered if a double surgery (extraction and implantation in the same patient) is not performed. Similarly, the high costs of the bone preparation, as well as sterilization treatments, must be taken into account before storage for clinical use [5]. On the other hand, another option for pathologies for bone regeneration is the use of xenogeneic drugs; however, there is a possibility of immunological reactions and disease transmission [6]. In recent years, the use of synthetic scaffolds for bone regeneration has been investigated to reduce these limitations [7,8].

**Figure 1:**
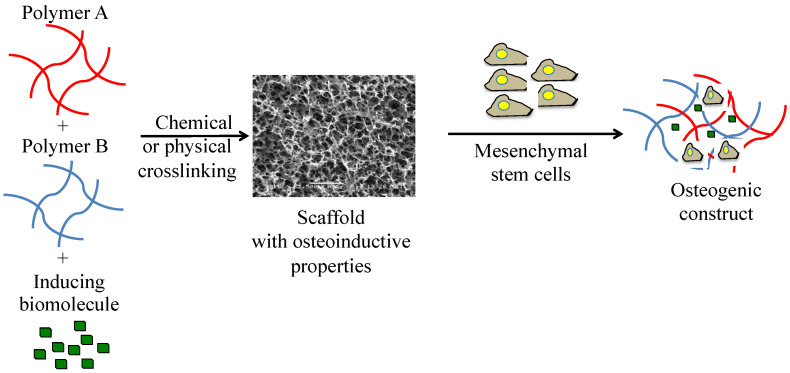
Polymeric scaffolds with osteoinductive properties seeded with mesenchymal stem cells to obtain an osteogenic tissue construct for cleft palate regeneration.

Currently, tissue engineering helps to a great extent in the regeneration of various pathologies that affect the human body, taking into account three fundamental parameters, namely polymeric biomaterials, cell lineage and nutrients or stimulating molecules (Figure 1). This contributes to reducing the effects of diseases and increases the success of reconstructions of the tissues of the affected areas, improving the living conditions of the affected patients. Bone repair is a key challenge in bone tissue engineering, as it requires an interconnected network with a proper pore organization. Similarly, some authors mention that the bone architecture, pores and their interconnections are necessary for vascularization of the tissues and for positive regeneration [9,10]. For this reason, the following review analyzes different biomaterials in which nutrients or biomolecules are added to improve the bioactive properties of the tissue construct in order to regenerate new bone.

## 2. Etiology

Nasopalatine cleft from an etiological point of view is considered a hereditary congenital pathology. It can be classified in a specific way as being due to (a) intrinsic or genetic factors and (b) environmental or extrinsic factors [11].

### 2.1. Environmental or Extrinsic Factors

In these types of infectious agents, metabolic and pharmacological agents are found. Among the infectious agents are congenital rubella and congenital toxoplasmosis, as well as cytomegalovirus infections and congenital syphilis, which have been associated as possible causes [12]. With regard to metabolic agents, these are linked to folic acid deficiency, gestational diabetes, zinc deficiency and maternal obesity [13]. Among pharmacological agents, the teratogenic effects of certain drugs associated with this disease can be listed, such as corticosteroids, benzodiazepines, anticonvulsants, thalidomide and antimetabolites, which antagonize the metabolism of folic acid. Alcohol and tobacco use during pregnancy have been mentioned as causal candidates for CP [14].

### 2.2. Genetic and Intrinsic Factors

Genetic factors are associated with complex syndromes. As shown below, 40% of clinical cases of CP have a genetic etiology. When parents without CP alterations have a baby with CP, there is a 4.4% chance that their second baby will develop CP. When the father is affected by CP, there is a 3.2% chance that his first child will develop CP, and if this happens the probability that CP will present in his second child increases to 17%.

With regard to the syndromic pathologies associated with CP, the following can be mentioned:

(1) Trisomy 13–14 or Patau’s syndrome. The pathology shows cardiac defects, mental retardation, impaired vision development, deafness, urogenital alterations and CP in 70–80% of cases [11];
(2) Trisomy 17–18 or Edwards’ syndrome. Alterations in renal function, bone and cardiac malformations, mandibular micrognathia, mental retardation, joint hypermobility, and CP are present in 15–17% of cases [11];
(3) Trisomy 21 or Down’s syndrome. There are abnormalities such as atrial dysplasia, mandibular prognathism and heart disease in 12% of cases, as well as mental retardation, macroglossia and CP in 6% of cases [11];
(4) Treacher Collins syndrome. Autosomal dominant disease not linked to sex, which is characterized by hypoplasias that damage the structures of the first and second branchial arch, zygomatic process of the temporal bone, maxilla, zygomatic bone and middle-external ears. Linear coloboma in the outer third of the lower eyelid can also be observed in 50% of cases, as well as antimongoloid obliquity. Similarly, 30% of cases are associated with CP [15];
(5) Van der Woude syndrome. Autosomal dominant disease not linked to sex, associated with deletion of chromosome 32 and characterized by CP, partial maxillary anodontia, pits in the lower lip (vermilion border) associated with minor salivary gland defects, ankyloglossia and alterations in the temporomandibular joint. In addition, other manifestations such as heart and lower limb malformations have been reported. This occurs in 1 in 100,000 births, while 2% of patients with CP are associated with this pathology [16];
(6) Velocardiofacial syndrome or Shprintzen–Goldberg syndrome. Autosomal dominant pathology associated with deletion of chromosome 22 characterized by CP, mental retardation and cardiac abnormalities, as well as non-constant ophthalmological, immunological, endocrine and orthopedic alterations [17];
(7) Robin sequence. Agglomerations of orofacial malformations characterized by glossoptosis, micrognathia and characteristic “u”-shaped CP. The complexity of the micrognathia can lead to upper airway obstructions [18].

## 3. Prevalence

Epidemiologically speaking, CP has an incidence rate of 20%. It should be mentioned that when this pathology occurs, it is related to systemic diseases or congenital malformations [19,20].

The world average ethnic incidence rates of live-born patients are presented below:

(a) Africans: 1:2500;
(b) Caucasians: 1:1000;
(c) Mexicans: 1:700;
(d) Latin Americans: 1:650;
(e) Asians: 1:500.

However, the secretary of health indicates its incidence as being between 1:600 and 1:200 births, depending on the country, racial group, sex and socioeconomic stratum in question [13,21,22].

Regarding the classification of the palatal cleft, this is a subject that generates controversy due to the different criteria used for its classification. Classification based on the anatomical description of the fissure and structures involved is the most accepted approach, with different forms of CP listed below:

(a) Cleft palate;
(b) Submucosal cleft palate;
(c) Velopharyngeal insufficiency;
(d) Robin sequence.

It is worth mentioning that in this classification process, it is necessary to specify certain characteristics that may occur:

- Primary: When the anterior part of the palate (premaxilla) is involved;
- Secondary: When the affected area is in the foramen.

## 4. Functional Aspects of the Bone Graft Healing Process and Bone Formation

The various aspects of bone formation depend on the composition of the graft and its origins, giving different regeneration results associated with the below properties [23].

Osteoconduction: This occurs in a “skeleton” that helps capillaries and precursor bone cells to develop, thereby creating a scaffold where bone can be created around it.

Osteogenesis: Osteoblasts derived from the graft contribute to the formation of new bone.

Osteoinduction: This involves the stimulation of osteoprogenitor cells that differentiate into osteoblasts.

An ideal material for bone regeneration must present osteogenic, osteoinduction and osteoconduction properties and must allow the stimulation of neoangiogenesis with mechanical stability and minimal morbidity, ideally with zero complications [24,25].

The histology of cortical and cancellous grafts plays an important role in their biological properties. Cortical grafts mainly provide stability; however, they have poor osteogenic capacity due to the slow bone growth caused by the prolonged resorption of materials [26].

Cancellous grafts stimulate osteogenesis due to the presence of osteoblasts, osteocytes and mesenchymal stem cells within their structures. A mixture of cortical and cancellous grafts can ensure stability and osteogenesis. There are also differences in mechanical strength, which increases in cancellous bones due to the faster deposition rate of new bone tissue, whereas in cortical grafts the strength decreases [27].

## 5. Clinical Therapy

Treatments for the reconstruction of damaged areas of CP are classified into two groups: surgical and non-surgical. Here, we will focus on surgical procedures, for which we will use tissue scaffolds as tools for bone regeneration. These treatments can be classified into two stages: primary and secondary [28].

The first includes the closure of the cleft palate (primary closure), as well as nasal surgical revision, which is usually performed within the first 5 years of the patient’s life. However, other studies have shown that an early closure can be performed after 6 months of the birth of the patient, since there is better muscle activity of the soft palate and consequently less scarring in the soft tissues of the CP. This is favorable, since a surgical intervention of the hard palate compromises the anteroposterior growth of the maxilla, which is suggested to be performed at 18 months of age [28].

The second surgical stage begins during the first 7 years of life and includes the management of fistulas, where bone defects lead to sequences of functional sequel. This is known as cleft palate sequel (CPS), giving origin to the main treatments for the reconstruction of bone defects through the use of bone grafts [11]. Research and the experiences of various authors have shown that autologous bone grafts present the results for bone reconstruction [26,28]. This superiority is mainly due to the ability of such grafts to integrate into the damaged area through the biological processes of osteogenesis, osteoconduction and osteoinduction.

Having been established through biological development and clinical evolution, the autologous bone graft approach is the primary choice for the reconstruction of a CP. However, the availability of autogenous bone presents certain drawbacks, particularly in pediatric patients where its extraction is forced, which may mean that the graft bone is not suitable for the reconstruction of the alveolar bone. Additionally, this graft process is invasive and has the possibility of producing complications at the donor site, while paresthesia, postoperative pain, infections and hearing difficulties may also occur [29,30].

## 6. Biomaterials Applied in Bone Regeneration

Certain biomaterials with applications for bone regeneration have been reported. Among them, poly(ε-caprolactone) and hydroxyapatite are the most used raw materials. Tissue constructs have also been designed using two or more components to enhance or improve the bioactive characteristics and properties, providing an architecture that encourages cell proliferation to create new tissue. Among the constituents of the biomaterials that have been studied are chitosan, calcium phosphate, magnesium, silk fibroin, polyvinyl alcohol, polyurethanes, bioactive glass, polymethylmethacrylate and polylactic coglycolic acid.

### 6.1. Polymer-Based Bone Substitutes

**Figure 2:**
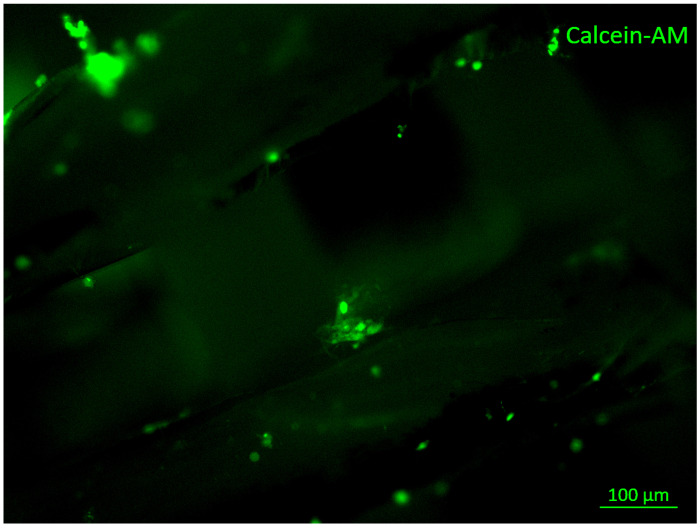
A 3D-printed PCL scaffold seeded with pig dental pulp stem cells using calcein–AM and live and dead assays (live assay shown in green) (courtesy of L.M. Rodríguez-Lorenzo from the Department of Polymeric Nanomaterials and Biomaterials, Institute of Science and Technology of Polymers (ICTP-CSIC, Madrid, Spain). The image is taken from a fluorescence microscope at 10× magnification.

Poly(ε-caprolactone), also known as PCL, can imitate the matrix of the bone tissue that is to be replaced, offering properties that favor its use in this application and in 3D scaffolds, where it generates a porous structure that provides a feasible environment for new bone tissue to be created [31,32]. An important characteristic of PCL is that it is soluble in a wide range of organic solvents, making it a promising material for research, as it can be mixed with various polymers to design composite biomaterials [33]. A previous study using PCL polymer elaborated the scaffold through the electrospinning process. Once the tissue support is obtained, it is coated with a layer of calcium phosphate to increase the osteoconduction in the material, which was shown to be satisfactory in bioactive tests [34]. Other studies have worked with PCL and carbon nanotubes, whereby the biomaterial provided an ideal three-dimensional microenvironment for the cells. The geometry of the scaffold and its bioactive properties show acceptable cellular biocompatibility and can be used for the fabrication of scaffolds to create bone tissue [35]. In the same way, Researchers manufactured a PCL (Figure 2) scaffold using the 3D printing method. The results showed that the morphology can be controlled using this technique, while cell viability tests and staining with von Kossa red and Aliazarin showed the binding, proliferation and differentiation capacity levels of swine dental pulp stem cells (DPSCs) in the obtained PCL scaffolds. These results indicate that these biomaterials have potential uses in additional models and preclinical experiments for bone engineering studies [36].

**Figure 3:**
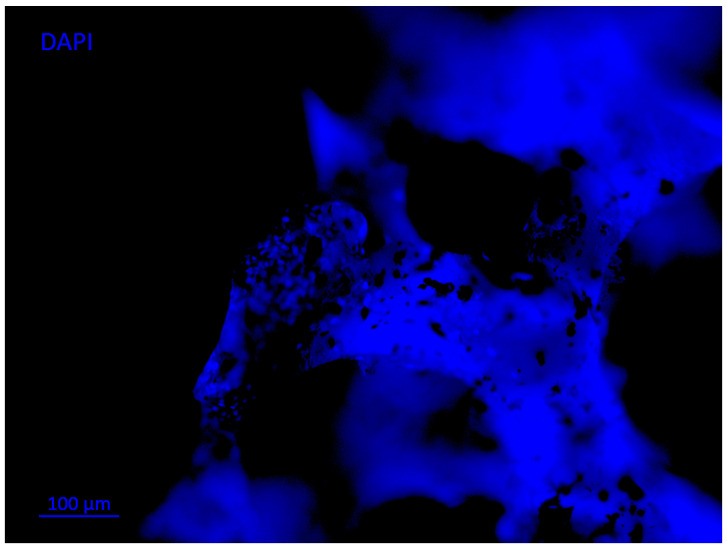
Polyurethane scaffold seeded with dental pulp stem cells (nuclear staining using DAPI). Image taken from a fluorescence microscope showing cell proliferation in the scaffold matrix for bone regeneration (courtesy of J.V. Cauich-Rodríguez from CICY, Mérida, Yucatán, México).

A PCL–tricalcium phosphate β (PCL–TCP) scaffold was produced via electrospinning, for which the ratio used was 80:20%. In the elaboration, it was decided to use TCP because it improves the rigidity and osteoconductivity of PCL-based scaffolds. Similarly, mesenchymal stem cells (MSC) and platelet-rich plasma (PRP) have been reported to stimulate bone formation by promoting osteoblast formation and vascularization. This is why bioactive tests were performed in the mentioned study. Regarding the results, the PCL–TCP scaffold proved to be biocompatible for bone regeneration in bone defects. Furthermore, the incorporation of MSC and PRP optimized the bone regeneration process with respect to the scaffold replacement rate, the height of the regenerated bone and the implant stability [37]. Recent research has shown that scaffolds made of PCL and hydroxyapatite (HA) allowed cells to adhere and proliferate into the pores of the structures that were designed. Likewise, a suitable environment was fostered to promote cell migration, differentiation and fluid circulation within the PCL–HA scaffold, making it a potential candidate for bone regeneration. The HA–PCL composite scaffolds were prepared via sol–gel method at room temperature [38]. Most polymer-based scaffolds are suitable for incorporating bioactive molecules and growth factors to potentially enhance the bioactive properties of the scaffold (Figure 3; biomaterials with osteoblast proliferation) [39,40]. The is done in order to imitate the matrix of the bone tissue to be replaced, offering osteoinductive properties and adequate structural porosity.

Recent research has involved the use of combinations of polymers for the preparation of scaffolds for bone grafts. In this approach, synthetic, natural and non-degradable polymers such as polymethylmethacrylate (PMMA) are combined with biodegradable polymers such as polylactic acid in order to take advantage of the polymer synergy [41,42]. On the other hand, PMMA powders with a mixture of liquid monomers can be easily molded to repair the damaged bone area or can be used in injectable methacrylate forms before polymerization [43,44]. A scaffold was also designed using the lyophilization technique with polymethylmethacrylate, which was combined with a platelet gel before being implanted in bone defects in rats. The results showed that the defect areas were filled with fibrocartilage tissue and several islands of calcified cartilage and HA crystals after scaffold implantation. Similarly, they concluded that the bone formation and bone volume were remarkable when this scaffold was used [45].

Polymer-based biomaterials have wide potential for use in tissue engineering, taking into account the dimensions of the micro- and macropores in the constructs as one of the most important structural characteristics [31,39]. This will largely determine the bioactive properties that the biomaterial will present, which will be necessary for adequate bone regeneration.

### 6.2. Hydroxyapatite

HA is a crystalline compound with a hexagonal lattice that has a specific formula [Ca_10_(PO_4_)_6_(OH)_2_], being the primary mineral constituent of teeth and bones [41]. As such, it is biocompatible and does not produce an inflammatory response [46,47]. HA is found in nature, forming porous structures that vary according to the bone site from which it is extracted; for example, trabecular bone has 65% porosity and measures 100–200 µm in diameter, allowing osteoinductive properties [48]. Another important characteristic is its slow reabsorption, which usually keeps the material in its initial condition for 2–3 years after implantation. This allows slow growth of the bone tissue with cell proliferation within the material [48,49]. HA also shows very good mechanical properties, with compressive strength values of up to 160 MPa, with applications in small areas of bone under low load conditions [48,50,51]. 

Recently it was shown that lyophilized co-membranes can serve as mineralization substrates to create osteconductive scaffolds. However, due to their low mechanical strength, it is necessary to mix them with another biomaterial such as a ceramic to help improve this property and to help regenerate the bone tissue [52,53]. Given the above, some investigations have involved the design of HA–collagen (co) composite polymeric materials, producing scaffolds with better differentiation of osteoblasts and with increased osteogenesis. Additionally, the mechanical properties are improved, unlike when using HA alone. This is because collagen fibers show ductility, which increases the fracture toughness of HA. In this research, the materials were formed via the compaction of powders by first selecting the particle size of the HA powder and then making the mixtures with the collagen [54].

One of the great challenges in research with HA is studying its bioactivity in relation to the concentration that the scaffold must contain in order to give the material osteogenic activity and to create bone tissue. Porous chitosan (cs) and hydroxyapatite (HA) scaffolds have been studied previously, whereby the researchers first synthesized hydroxyapatite nanoparticles (nHAp) in the presence of hyperbranched polyethyleneimine to control the size and morphology of hydroxyapatite crystals. They then prepared a 3% *w*/*w* qs solution in acetic acid and added nHAp at a 75:25 cs weight ratio. The resulting suspension was then mixed and lyophilized to obtain the three-dimensional scaffolds. The results showed high bioactivity, high cell adhesion and good proliferation, as well as a well-disseminated cell distribution within the osteogenic scaffold structure [55]. Similarly, in another study, the same researchers made a porous chitosan–HAp scaffold, only this time using the porogenic leaching method. The cs–HAp mixture had an 80:20 ratio and the NaCl particles (particle sizes in the range of 100–300 µm) were homogeneously mixed at weight ratios of 1:1, 1:5, 1:10 and 1:20. The NaCl particles were then subsequently removed from the scaffolds via extraction in cold water (20 °C), followed by extraction in warm water (50 °C) for approximately 12 h each. The histological results showed that the cs–HAp scaffold did not show inflammation in the area where it was implanted and that new bone formation was found around the scaffold in vivo. This result indicated that inflammation due to chitosan can be reduced by forming a homogeneous chitosan–HAp compound, improving the osteoconductivity [56].

In another investigation, cs–HA scaffolds obtained using the lyophilization technique were designed. In the same way, the researchers incorporated heparin as one more component in the scaffold. The cs–HA–heparin scaffold showed no cytotoxic effects, sustained heparin release and favorable angiogenic responses [57]. Has also been published an extensive review on the molecular interactions of heparin and heparan sulfate proteoglycans related to revascularization. In this review, the authors highlighted the ability of heparin to bind with angiogenic growth factors (AGF), including both inhibitors and promoters. The balance between these two is how heparin helps regulate neovascularization [58].

Research has also been performed on scaffolds composed of nHAp–co–poly(lactic-co-glycolic acid)–PLGA–graphene oxide. In this study, tissue scaffolds were developed through the lyophilization process, resulting in synthesized scaffolds with an interconnected three-dimensional porous structure. Furthermore, graphene oxide was found to improve the hydrophilicity of scaffolds and increase their mechanical strength. Cell adhesion and proliferation of osteoblastic cells in the designed scaffold were also observed in in vitro experiments, facilitating cell adsorption, growth and proliferation in the scaffold and making it promising for applications in bone defects [59]. The foregoing could be promising for applications in bone tissue defects. In another investigation, a biomaterial was also designed using PLGA with a co–HA coating, which was elaborated via the electrospining method, with the results showing a significant improvement in the activity of alkaline phosphatase, indicating that the co–HA coating compound played an important role in controlling the bioactivity of PLGA-based scaffolds. These studies have clearly indicated that the co–HA combination is useful in providing a favorable environment for monitoring the biological activity of scaffolds [60].

**Figure 4:**
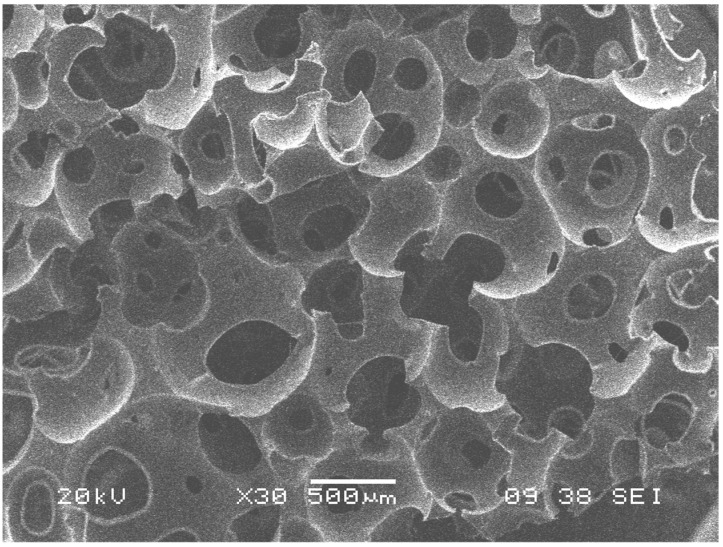
SEM micrograph: structure of a PU–titanium particle scaffold with interconnected pores, which promotes cell proliferation towards the interior of the scaffold (courtesy of Fernando Javier Aguilar-Perez from CICY, Mérida, Yucatán, Mexico).

Investigations of foam materials based on HA (hydroxyapatite) and segmented polyurethanes (PU) were performed with glutamine or ascorbic acid as chain extenders, with biocompatibility studies revealing that the materials containing ascorbic acid allowed an increase in the proliferation of alveolar osteoblasts [61]. Another study of PU and HA with 30% HA by weight showed a porosity above 60% with the presence of pores measuring 100–800 µm, which were within the parameters for the regeneration of bone tissue. In this study, gradual in vitro degradation was observed, which was considered clinically adequate [62]. PU materials have also been designed with bioactive compounds such as β-glycerophosphate, as well as ascorbic acid and dexamethasone, which provide in vitro and in vivo improvements for bone formation and can be used for bone tissue engineering. To obtain the porous scaffolds, the foaming technique was used, in which constructs with a three-dimensional geometry with characteristics suitable for bone tissue engineering were used [63]. Another investigation carried out a study on PU foams, to which titanium particles were added to see if there were any changes in the bioactive properties that the material would present. The structure presented by the scaffold was a matrix with interconnected pores, as shown in Figure 4. This system had beneficial effects on the viability of stem cells from human dental pulp and osteoblast cells [64].

### 6.3. Calcium Phosphate Cements

Calcium phosphate cements (CPC) were first used by Brow Chow in 1986, later being approved by the FDA in 1996 for the treatment of bone defects in the absence of loading [41,65]. This material is bioabsorbable and can be modified in its formulation to improve the structural composition, which can delay absorption by up to 2 years after implantation [66]. The osteoconductivity of the material is given in the isothermal hardening reaction, which varies from 15 to 85 min, depending on the formulation, and is caused by the nanocrystals that are formed [67]. The most important thing about this biomaterial is that the paste can be shaped so that it is implanted into the affected area, avoiding spaces between the bone and the biomaterial. [41]. Similarly, there are already CPCs that have been used in injection presentations for minimally invasive procedures. Additionally, HA-based calcium sulfate biomaterials have arisen because CPCs by themselves are fragile and can lead to complications, with infections occurring in 5% and general complications in 13% of cases [68]. Another study evaluated the efficiency of the local administration of bisphosphonates by injectable CPC in vertebral bodies of the lumbar spine of an osteoporotic sheep model, whereby the consequences of osteoporotic fractures were highly detrimental in the trials. This cement combined with bisphosphonates in vertebral body bone defects had a beneficial impact on both the bone content and the microarchitectural properties of the trabecular bone surrounding the implant. These animal studies showed promise for the use of injectable CPCs for bone repair and regeneration [69].

In addition, ceramic materials are fragile, with low mechanical resistance and low fracture toughness, meaning they cannot be used as the only material for the manufacture of scaffolds [70]; however, they can be used to improve the bioactive and mechanical properties of scaffolds for bone regeneration.

### 6.4. Other Biomaterials Used for Bone Regeneration

Another biomaterial used for bone regeneration is silk fibroin (SF). In a previous study, the researchers elaborated the scaffold using the electroylation technique, which was then cross-linked with glutaraldehyde and the osteoinductive recombinant human bone morphogenic protein-2 (rhBMP2) was added in order to evaluate the osteoinductive potential of the resulting scaffold. A homogeneous structural morphology with good mechanical properties was found, while the results of the bioactive tests showed biocompatibility within the acceptable ranges for applications related to bone tissue engineering [71]. Similarly, in another investigation, we worked with SF–cs nanofiber scaffolds (50:50%, using the electrospinning technique to elaborate the three-dimensional scaffolds. The results in this investigation showed osteogenic proliferation and differentiation within the scaffold, making it a promising candidate as a composite construct for bone tissue engineering [72].

Similarly, magnesium (Mg) is a biomaterial that has been widely used in the elaboration of constructs for bone regeneration. However, due to the corrosion reaction, which accelerates hydrogen formation leading to premature loss of mechanical strength and the production of harmful hydrogen gas, it has been used as a compound to minimize this problem [73]. Investigations have also been performed on designs involving Mg materials with HA and bioactive glass as construct fillers. The results obtained from the study of Mg matrix compounds showed improvements in the degradation and bioactivity properties of the scaffold. In this study, the scaffolds were formed by mixing the magnesium alloy with respect 20% HA by weight after extrusion at 400 °C, thereby obtaining the tissue constructs in the form of cylinders [74]. Bioactive glass (45S5) and magnesium composite materials have also been developed and improvements in bone formation have been found following the proliferative stimulation of osteoblasts. In this investigation, composites containing ZK30 alloy as the matrix and 45S5 bioactive glass (BG) as the reinforcement phase were fabricated using a semi-solid casting (SSC) method and a powder metallurgy (P/M) method [75,76]. Similarly, bioglass (10% *w*/*v*) has been added as an osteoconductor in cs scaffolds along with polyvinyl alcohol (PVA) at a 3:7 ratio, whereby the scaffolds were manufactured using the lyophilization method. Favorable results were obtained, showing osteogenic potential, whereby new bone was formed in the peripheral margins of the defect [77].

## 7. Biomaterials Applied in the Regeneration of the Cleft Palate

As an alternative, one of the strategies used in tissue engineering involves implanting biomaterials to favor the regeneration of the affected tissue [78]. These biomaterials have been developed mostly in the form of compounds or mixtures of ceramics and polymers in order to create ideal scaffolds with synergy between the components, which increases the biological properties of the tissue construct.

To date, the biomaterials used to regenerate areas of the CP have been designed based on single-layer tissues, focusing primarily on the granules [79]. However, the alveolar cleft pathology is characterized by a defect in which two walls are involved: the two layers of the nasal floor and the continuous oral mucosa. Therefore, the granules of the biomaterials hinder the mechanical and dimensional stability of the reconstructed areas, causing difficulties in healing and provoking possible infections [80]. To help mitigate this problem and make the reconstruction of the CP more sophisticated, 3D structures with similarity to the bone to be repaired have been considered, and their bioactive and mechanical properties have been studied based on the osteogenic effect of the designed material [11]. 

In another study, designed a scaffold with biodegradable calcium-phosphate-based biomaterials using the 3D printing technique, which allows the manufacture of bone grafts for tissue engineering in the affected areas. The constructs presented two different orientations of the fibers, with rotations of 60º and 30º. These calcium phosphate scaffolds were applied to fill the artificial bone defects in the palate of adult Lewis rats, showing good support and excellent osteoconductive properties. It was also shown that the geometry of the pores significantly influences bone formation, since the 60° fiber rotation formed triangular pores with significantly better performance than the 30° fiber rotation. However, the scaffold degradation test did not show any changes, indicating the need for further development of the material, either by modifying the cement matrix with porogens or by varying the composition of the matrix towards more soluble phases [81].

On the other hand in Singapore polylactic acid (PLGA) bioscaffold was used, in which bone marrow mesenchymal stem cells (BMSC) were seeded. A non-syndromic cleft palate animal model was prepared, for which albino Wistar rats were selected. The results show that the control group in which BMSC were not seeded did not show closure of the palatal defect, while the group of rats who received the PLGA–BMSC scaffold presented normal healing that completely closed the defect. This is why the scaffolds developed in this study mimicked the extracellular matrix, allowing the interconnectivity of the obtained pores, which induced cell adhesion and migration [82]. Similarly, another study involved PLGA, in which the authors used the electrospinning method to obtain the scaffolds. They made use of mesenchymal stem cells (MSC) in the scaffolds, then these tissue constructs were implanted in a juvenile porcine alveolar cleft model with a critical size defect close to 1.7 cm. The results of the studies showed bone formation in the surgically created alveolar cleft defect. Therefore, the bioactive properties of these scaffolds were favorable and proved that the strategy of using stem cells in the scaffold leads to greater treatment efficacy [83].

Has also worked on an alveolar cleft model using polyphosphate polymer (PolyP), a physiological polymer made up of orthophosphate units linked together by phosphate bonds. This compound exists naturally in platelets, while microparticles in the form of calcium (Ca–PolyP) were added to functionalize the compound. This PolyP biomaterial presents primary active compounds and involves the regeneration of tissues via osteogenic factors, which in the research generated an adequate quantity and quality of bone formation [84].

Other researchers elaborated a three-dimensional scaffold with a cement based on α-tricalcium phosphate (CPC). Unlike liquid calcium phosphate cements in powder form, in this CPC formulation the cement consisted of a mixture of precursor powders and a hydrophobic but biocompatible liquid vehicle, which was exchanged with water after implantation, thereby initiating the setting process. This allowed for unlimited injectability through a nozzle and 3D printing fabrication. Then, fibrin with human mesenchymal stem cells (hMSC) was added to infiltrate the pores between the CPC strands to distribute the cells evenly within the three-dimensional scaffold structure. The results showed that the macroporous CPC structure formed using 3D printing filled with the fibrin hydrogel with cells and accelerated the formation of bone tissue in vivo. This is due to the rapid degradation of fibrin and the migration of embedded cells towards the adjacent CPC strands, followed by their adherence to the surfaces. This efficiently and homogeneously places the cells in the alveolar cleft, where they promote the formation of new bone and the healing of the bone defect [85]. Another investigation compared CPC against a biomaterial composed of HA and CPC (60:40%), known commercially as a Maxresorb injection (Botiss Biomaterials), in order to evaluate the pattern of bone regeneration and quantify the new bone after grafts in alveolar cleft defects in rabbits. The results of this study demonstrated the presence of new bone substitute materials after bone formation and grafting. In addition, the alveolar cleft defect was repaired with a higher residual graft volume and bone volume fraction when evaluating the HA–CPC composite biomaterial compared to CPC [86].

Gel-shaped scaffolds have also been based on alginate seeded with allogeneic mesenchymal stem cells derived from bone marrow (BM-MSC). These biomaterials were placed in surgically created and critically sized cleft palate defects in rats. Relevant results were shown by histological analysis of the area of the cleft palate in the rats, whereby the addition of BM-MSC stimulated bone formation in the center of the implant compared to the scaffold that did not contain BM-MSC, which only created bone around the defect. This demonstrated the potential of this new biomaterial for use as a bone substitute graft material for cleft palate repair. In addition, the biocompatibility of the alginate-based hydrogel and the actual benefits for tissue healing were verified [87].

Researchers designed a an absorbable collagen sponge (ACS) loaded with bone morphogenetic protein 2 (BMP-2) with added nanofibers (NF) to maintain the release of BMP-2 and improve the properties of the bone graft. The results of a rodent cleft palate model showed higher percentages of bone filling (% GC) at the defect sites in scaffolds made of NF–ACS–BMP-2. This confirmed the good osteoinductive properties of the tissue construct, making it a promising scaffold for the delivery of BMP-2 used in the reconstruction of the cleft palate [88].

In another study, a tissue engineering scaffold for cleft palate repair was designed in rats using a mixture of poly(1,8-octamethylene citrate) (POC) with a decellularized scaffold membrane (DSM–POC). This was achieved with different layers to form films and to remove the solvent at room temperature. The results showed a certain level of collapse of the structure and less rigidity. However, the material was biocompatible with mesenchymal stem cells, while the scaffold also showed adequate biocompatibility with almost complete hard and soft tissue healing, and also showed minimal interference with the natural growth and development of the rat palate. Similarly, a good material was obtained that could improve and promote the regeneration of hard and soft tissues with craniomaxillofacial congenital defects [89].

Current research developed a new tissue engineering protocol to obtain a non-immunogenic mucoperiosteal scaffold suitable for allogeneic transplantation and cleft palate repair with in vitro revascularization with human bone marrow-derived mesenchymal stem cells (hBM-MSC). This procedure uses a microperforation based on quantum molecular resonance (QMR), which improves the osteogenic differentiation of the hMSC. The results showed that the collagen mesh was preserved, causing an increase in the osteoinductive potential of the mesenchymal precursor cells. This scaffold has a potential impact on palatal bone regeneration, which could lead to future clinical applications in humans [90].

The closure of an oronasal fistula was also presented in another study, where a collagen (co) resorbable membrane was used as a tissue scaffold for repair in three layers to avoid recurrences. These interpositional grafts provided a scaffold for tissue growth, revascularization and mucosal epithelialization. This scaffold was placed in a 27-year-old male patient and was firmly fixed on the repaired nasal layer. The successful results were attributed to the wrapping of the collagen membrane around the repaired nasal layer, which was fixed in place until graft integration occurred. The collagen membrane selectively guided the regeneration of the oral mucosa and also provided a barrier or resistance to wound rupture [91]. It has also been designed. a collagen–hydroxyapatite biomaterial for the reconstruction of the palate area in humans. The results showed uncomplicated bone formation at the donor site 6 months after the operation. This bone regeneration was due to the osteoinductive and osteoconductive properties of the graft material used. Furthermore, this membrane showed promising advances that overcame the drawbacks of the standard autogenous bone graft method [92].

Other researchers developed a biomimetic multilayer palate substitute for bone tissue and oral mucosa applications using rabbit cells and biomaterials. They used a nanotechnology technique based on plastic compression, placing several layers of nylon mesh with a 0.22 µm pore size on top to protect the epithelium from compression. This graft was placed in defects in rabbits, with the results showing that the design of the multilayer 3D tissue scaffold is capable of integrating into the host tissue and contributing to the normal development of the palate, since the in vivo graft was able to induce cell and tissue differentiation [79].

Another work used a PDO (polydioxanone) matrix to repair areas of cleft palate defects in humans. The PDO matrix fully fitted under the palatal flap and was fixed with absorbable screws during the healing phase. This contributed to minimal postoperative morbidity and a high recovery rate in the affected area. The results showed complete healing at 6 months in all cases, while the fistula did not persist or recur [93]. In the same way, the PDO was used alone or combined with nanohydroxyapatite (nHA) and fibrinogen (Fg) with different simulated body fluids (SBF), with the scaffold being obtained via electrospinning. The addition of Fg resulted in smaller fiber scaffolds with thin sheet mineral deposition, unlike the individual fiber mineralization seen in PDO and PDO–nHA scaffolds. The study showed the development of a mineralized porous nanofibrous scaffold for cleft palate repair with high potential to help with the induction of three-dimensional bone formations in defects as effective substrates [94].

On the other hand, scaffolds of PCL have been elaborated using the electrospinning technique, with the results showing good biocompatibility and cell binding and also allowing osteogenic differentiation of human osteogenic progenitor cells. Human-jaw-derived cells (hJPC) could be a new source of cells for cleft palate repair, which could be obtained from palate closure surgery at a patient age of approximately 1 year. This would also be a cost-effective and easily scalable technique for stimulation and repair of cleft palate and other oral maxillofacial reconstructions, such as alveolar ridge augmentation for tooth implantation and guided bone tissue regeneration for periodontal surgery [95].

In recent work, it has been designed a scaffold using the lyophilization technique by creating an epigallocatechin gallate (EGCG) modified gelatin sponge in a rat congenital cleft jaw model. The study looked at the revascularization of the damaged area with the use of multipotent progenitor cells, such as adipose-derived stem cells (ADSC) and dedifferentiated fat cells (DFAT). In addition, bone formation was enhanced in defects 4 weeks after the scaffold was implanted. The results showed that these cells successfully induced better bone formation, and in those scaffolds that used EGCG there was greater bone formation than in those that did not contain it. This suggests that EGCG induces the differentiation of mesenchymal stem cells into osteoblast cells [96].

## 8. Conclusions and Future Perspectives

In recent years, a large number of materials have been used to help in the reconstruction of bone tissue in areas of the cleft palate. Some of these are products of synthetic and biological materials. The recent research has focused on summarizing the developed scaffolds with bone regeneration properties that have potential for application in cleft palate regeneration. In the same way, studies have already been performed on the design and manufacture of polymeric scaffolds with desirable porosity, complemented in some cases by bioactive molecules with osteoinductive properties to promote the desired vasculature and the formation of new bone in areas of the cleft palate. Among the most promising polymeric materials for bone tissue engineering that we have found are hydroxyapatite and collagen, as they are components of bone in its natural form. However, the concentrations in the constructs of these materials greatly modify the final bioactivity of the tissue construct. Similarly, in the elaboration of the scaffolds, a mixture of 2 or more biomaterials can be used to obtain constructs with ideal bioactive and physicochemical properties that will benefit from better regeneration of damaged bone tissue. However, mixtures of two or more polymers and the presence of functional groups in the chemical structures of any of the polymers being used will reduce the final potential of the tissue construct.

## References

[1] Becker D.B., Coalson R.S., Sachanandani N.S., Fair D., Lugar H.M., Kirchner L.E., Schlaggar B.L., Kane A.A. (2008). Functional neuroanatomy of lexical processing in children with cleft lip and palate. Plast. Reconstr. Surg..

[2] Montaño López A., Rincón Rodríguez H., Landa Solís C.I. (2012). Revista odontológica mexicana nasoalveolar bone graft integration range in patients with cleft lip and palate sequels. Medigraphic.

[3] Chigurupati R. (2012). Cleft Lip and Palate: Timing and approaches to reconstruction. Current Therapy in Oral and Maxillofacial Surgery.

[4] Gunzburg R., Szpalski M., Passuti N., Aebi M. The Use of Bone Substitutes in Spine Surgery: A State of the Art Review. https://link.springer.com/book/9783540426875.

[5] Saikia K.C., Bhattacharya T.D., Bhuyan S.K., Talukdar D.J., Saikia S.P., Jitesh P. (2008). Calcium phosphate ceramics as bone graft substitutes in filling bone tumor defects. Indian J. Orthop..

[6] Laurencin C.T., El-Amin S.F. (2008). Xenotransplantation in orthopaedic surgery. J. Am. Acad. Orthop. Surg..

[7] Evaniew N., Tan V., Parasu N., Jurriaans E., Finlay K., Deheshi B., Ghert M. (2013). Use of a calcium sulfate-calcium phosphate synthetic bone graft composite in the surgical management of primary bone tumors. Orthopedics.

[8] Kirkpatrick J.S., Cornell C.N., Hoang B.H., Hsu W., Watson J.T., Watters W.C., Turkelson C.M., Wies J.L., Anderson S. (2010). Bone void fillers. J. Am. Acad. Orthop. Surg..

[9] Tian T., Zhang T., Lin Y., Cai X. (2018). Vascularization in Craniofacial Bone Tissue Engineering. J. Dent. Res..

[10] Mercado-Pagán Á.E., Stahl A.M., Shanjani Y., Yang Y. (2015). Vascularization in Bone Tissue Engineering Constructs. Ann. Biomed. Eng..

[11] Kazemi A., Stearns J.W., Fonseca R.J. (2002). Secondary grafting in the alveolar cleft patient. Oral Maxillofac. Surg. Clin. N. Am..

[12] Wyszynski D.F., Beaty T.H. (1996). Review of the role of potential teratogens in the origin of human nonsyndromic oral clefts. Teratology.

[13] Shkoukani M.A., Lawrence L.A., Liebertz D.J., Svider P.F. (2014). Cleft palate: A clinical review. Birth Defects Res. Part C Embryo Today Rev..

[14] Cohen M.M. (2000). Etiology and Pathogenesis of Orofacial Clefting. Oral Maxillofac. Surg. Clin. N. Am..

[15] Bergland O., Semb G., Aabyholm F.E. (1986). Elimination of the residual alveolar cleft by secondary bone grafting and subsequent orthodontic treatment. Cleft Palate J..

[16] Reinoso Quezada S.J., Moscoso Mesías M. (2020). Case report Síndrome de Van der Woude. Rev. Fac. Odontol..

[17] Kernahan D.A. (1971). The striped Y—a symbolic classification for cleft lip and palate. Plast. Reconstr. Surg..

[18] Brusati R., Garattini G. (2000). The Early Secondary Gingivoperiosteoplasty. Oral Maxillofac. Surg. Clin. N. Am..

[19] Marx R.E. (1993). Philosophy and Particulars of Autogenous Bone Grafting. Oral Maxillofac. Surg. Clin. N. Am..

[20] Opitz C., Meier B., Stoll C., Subklew D. (1999). Radiographic evaluation of the transplant bone height in patients with clefts of the lip/alveolus/palate after secondary bone grafting. J. Orofac. Orthop..

[21] Parada C., Chai Y. (2012). Roles of BMP Signaling Pathway in Lip and Palate Development. Front. Oral Biol..

[22] Dixon M.J., Marazita M.L., Beaty T.H., Murray J.C. (2011). Cleft lip and palate: Understanding genetic and environmental influences. Nat. Rev. Genet..

[23] Bauer T.W., Muschler G.F. (2000). Bone Graft Materials: An Overview of the Basic Science. Clin. Orthop. Relat. Res..

[24] de Grado G.F., Keller L., Idoux-Gillet Y., Wagner Q., Musset A.M., Benkirane-Jessel N., Bornert F., Offner D. (2018). Bone substitutes: A review of their characteristics, clinical use, and perspectives for large bone defects management. J. Tissue Eng..

[25] Janicki P., Schmidmaier G. (2011). What should be the characteristics of the ideal bone graft substitute? Combining scaffolds with growth factors and/or stem cells. Injury.

[26] Titsinides S., Agrogiannis G., Karatzas T. (2019). Bone grafting materials in dentoalveolar reconstruction: A comprehensive review. Jpn. Dent. Sci. Rev..

[27] Roden R.D. (2010). Principles of Bone Grafting. Oral Maxillofac. Surg. Clin. N. Am..

[28] Guo J., Li C., Zhang Q., Wu G., Deacon S.A., Chen J., Hu H., Zou S., Ye Q. (2011). Secondary bone grafting for alveolar cleft in children with cleft lip or cleft lip and palate. Cochrane Database Syst. Rev..

[29] Berger M., Probst F., Schwartz C., Cornelsen M., Seitz H., Ehrenfeld M., Otto S. (2015). A concept for scaffold-based tissue engineering in alveolar cleft osteoplasty. J. Cranio Maxillofac. Surg..

[30] Al-Ahmady H.H., Abd Elazeem A.F., Bellah Ahmed N.E.M., Shawkat W.M., Elmasry M., Abdelrahman M.A., Abderazik M.A. (2018). Combining autologous bone marrow mononuclear cells seeded on collagen sponge with Nano Hydroxyapatite, and platelet-rich fibrin: Reporting a novel strategy for alveolar cleft bone regeneration. J. Cranio-Maxillofac. Surg..

[31] Wagner Q., Offner D., Idoux-Gillet Y., Saleem I., Somavarapu S., Schwinté P., Benkirane-Jessel N., Keller L. (2016). Advanced nanostructured medical device combining mesenchymal cells and VEGF nanoparticles for enhanced engineered tissue vascularization. Nanomedicine.

[32] Eap S., Ferrand A., Mendoza Palomares C., Hébraud A., Stoltz J.F., Mainard D., Schlatter G., Benkirane-Jessel N. (2012). Electrospun nanofibrous 3D scaffold for bone tissue engineering. Bio-Med. Mater. Eng..

[33] Porter J.R., Henson A., Popat K.C. (2009). Biodegradable poly(ε-caprolactone) nanowires for bone tissue engineering applications. Biomaterials.

[34] Brown T.D., Slotosch A., Thibaudeau L., Taubenberger A., Loessner D., Vaquette C., Dalton P.D., Hutmacher D.W. (2012). Design and Fabrication of Tubular Scaffolds via Direct Writing in a Melt Electrospinning Mode. Biointerphases.

[35] Valadez-González A., Rosales-Ibáñez R., Rodríguez-Navarrete A., Villamar-Duque T.E., Cano-Brown J., Carrillo-Escalante H.J., Ortiz-Fernández A., Hernández-Sánchez F. (2021). Tailoring surface properties of carbon nanofibers via oxidation and its influence on dental pulp stem cell viability of PCL/CNF composites. Polym. Bull..

[36] Rosales-Ibáñez R., Cubo-Mateo N., Rodríguez-Navarrete A., González-González A.M., Villamar-Duque T.E., Flores-Sánchez L.O., Rodríguez-Lorenzo L.M. (2021). Assessment of a PCL-3D printing-dental pulp stem cells triplet for bone engineering: An in vitro study. Polymers.

[37] Almansoori A.A., Kwon O.-J., Nam J.-H., Seo Y.-K., Song H.-R., Lee J.-H. (2021). Mesenchymal stem cells and platelet-rich plasma-impregnated polycaprolactone-β tricalcium phosphate bio-scaffold enhanced bone regeneration around dental implants. Int. J. Implant Dent..

[38] D’Antò V., Raucci M.G., Guarino V., Martina S., Valletta R., Ambrosio L. (2016). Behaviour of human mesenchymal stem cells on chemically synthesized HA-PCL scaffolds for hard tissue regeneration. J. Tissue Eng. Regen. Med..

[39] Eap S., Keller L., Schiav J., Huck O., Jacomine L., Fioretti F., Gauthier C., Sebastian V., Schwinté P., Benkirane-Jessel N. (2015). A living thick nanofibrous implant bifunctionalized with active growth factor and stem cells for bone regeneration. Int. J. Nanomed..

[40] Ferrand A., Eap S., Richert L., Lemoine S., Kalaskar D., Demoustier-Champagne S., Atmani H., Mély Y., Fioretti F., Schlatter G. (2014). Osteogenetic properties of electrospun nanofibrous PCL scaffolds equipped with chitosan-based nanoreservoirs of growth factors. Macromol. Biosci..

[41] Campana V., Milano G., Pagano E., Barba M., Cicione C., Salonna G., Lattanzi W., Logroscino G. (2014). Bone substitutes in orthopaedic surgery: From basic science to clinical practice. J. Mater. Sci. Mater. Med..

[42] Nandi S.K., Roy S., Mukherjee P., Kundu B., Basu D. (2010). Orthopaedic applications of bone graft & graft substitutes: A review. Indian J. Med. Res..

[43] Hernigou P., Ma W. (2001). Open wedge tibial osteotomy with acrylic bone cement as bone substitute. Knee.

[44] Laurencin C., Khan Y., El-Amin S.F. (2006). Bone graft substitutes. Expert Rev. Med. Devices.

[45] Oryan A., Alidadi S., Bigham-Sadegh A., Moshiri A. (2018). Healing potentials of polymethylmethacrylate bone cement combined with platelet gel in the critical-sized radial bone defect of rats. PLoS ONE.

[46] Ghosh S.K., Nandi S.K., Kundu B., Datta S., De D.K., Roy S.K., Basu D. (2008). In Vivo response of porous hydroxyapatite and β-tricalcium phosphate prepared by aqueous solution combustion method and comparison with bioglass scaffolds. J. Biomed. Mater. Res. Part B Appl. Biomater..

[47] Nandi S.K., Kundu B., Ghosh S.K., De D.K., Basu D. (2008). Efficacy of nano-hydroxyapatite prepared by an aqueous solution combustion technique in healing bone defects of goat. J. Vet. Sci..

[48] Okazaki A., Koshino T., Saito T., Takagi T. (2000). Osseous tissue reaction around hydroxyapatite block implanted into proximal metaphysis of tibia of rat with collagen-induced arthritis. Biomaterials.

[49] Daculsi G. (1998). Biphasic calcium phosphate concept applied to artificial bone, implant coating and injectable bone substitute. Biomaterials.

[50] Johnson K.D., Frierson K.E., Keller T.S., Cook C., Scheinberg R., Zerwekh J., Meyers L., Sciadini M.F. (1996). Porous ceramics as bone graft substitutes in long bone defects: A biomechanical, histological, and radiographic analysis. J. Orthop. Res..

[51] Spivak J.M., Hasharoni A. (2001). Use of hydroxyapatite in spine surgery. Eur. Spine J..

[52] Nudelman F., Lausch A.J., Sommerdijk N.A.J.M., Sone E.D. (2013). In Vitro models of collagen biomineralization. J. Struct. Biol..

[53] Strauss F., Kuchler U., Kobatake R., Heimel P., Tangl S., Gruber R. (2021). Acid bone lysates reduce bone regeneration in rat calvaria defects. J. Biomed. Mater. Res. Part A.

[54] Xie J., Baumann M.J., McCabe L.R. (2004). Osteoblasts respond to hydroxyapatite surfaces with immediate changes in gene expression. J. Biomed. Mater. Res. Part A.

[55] Chatzipetros E., Damaskos S., Tosios K.I., Christopoulos P., Donta C., Kalogirou E.-M., Yfanti Z., Tsiourvas D., Papavasiliou A., Tsiklakis K. (2021). The effect of nano-hydroxyapatite/chitosan scaffolds on rat calvarial defects for bone regeneration. Int. J. Implant Dent..

[56] Kashiwazaki H., Kishiya Y., Matsuda A., Yamaguchi K., Iizuka T., Tanaka J., Inoue N. (2009). Fabrication of porous chitosan/hydroxyapatite nanocomposites: Their mechanical and biological properties. Biomed. Mater. Eng..

[57] Nájera-Romero G.V., Yar M., Rehman I.U. (2020). Heparinized chitosan/hydroxyapatite scaffolds stimulate angiogenesis. Funct. Compos. Mater..

[58] Chiodelli P., Bugatti A., Urbinati C., Rusnati M. (2015). Heparin/Heparan Sulfate Proteoglycans Glycomic Interactome in Angiogenesis: Biological Implications and Therapeutical Use. Molecules.

[59] Liang C., Luo Y., Yang G., Xia D., Liu L., Zhang X., Wang H. (2018). Graphene Oxide Hybridized nHAC/PLGA Scaffolds Facilitate the Proliferation of MC3T3-E1 Cells. Nanoscale Res. Lett..

[60] Kwon G.-W., Gupta K.C., Jung K.-H., Kang I.-K. (2017). Lamination of microfibrous PLGA fabric by electrospinning a layer of collagen-hydroxyapatite composite nanofibers for bone tissue engineering. Biomater. Res..

[61] Cetina-Diaz S.M., Chan-Chan L.H., Vargas-Coronado R.F., Cervantes-Uc J.M., Quintana-Owen P., Paakinaho K., Kellomaki M., Di Silvio L., Deb S., Cauich-Rodríguez J.V. (2014). Physicochemical characterization of segmented polyurethanes prepared with glutamine or ascorbic acid as chain extenders and their hydroxyapatite composites. J. Mater. Chem. B.

[62] Luo K., Wang L., Wang Y., Zhou S., Zhang P., Li J. (2020). Porous 3D hydroxyapatite/polyurethane composite scaffold for bone tissue engineering and its in vitro degradation behavior. Ferroelectrics.

[63] Wang C., Cao X., Zhang Y. (2017). A novel bioactive osteogenesis scaffold delivers ascorbic acid, β-glycerophosphate, and dexamethasone in vivo to promote bone regeneration. Oncotarget.

[64] Aguilar-Perez F.J., Vargas-Coronado R., Cervantes-Uc J.M., Cauich-Rodriguez J.V., Rosales-Ibañez R., Pavon-Palacio J.J., Torres-Hernandez Y., Rodriguez-Ortiz J.A. (2018). Preparation and characterization of titanium—segmented polyurethane composites for bone tissue engineering. J. Biomater. Appl..

[65] Brown W.E., Brown P.W. (1986). A new calcium phosphate water-setting. Cements Research Progress.

[66] Russell T.A., Leighton R.K. (2008). Comparison of autogenous bone graft and endothermic calcium phosphate cement for defect augmentation in tibial plateau fractures. A multicenter, prospective, randomized study. J. Bone Jt. Surg. Ser. A.

[67] Burguera E.F., Xu H.H.K., Weir M.D. (2006). Injectable and rapid-setting calcium phosphate bone cement with dicalcium phosphate dihydrate. J. Biomed. Mater. Res. Part B Appl. Biomater..

[68] Afifi A.M., Gordon C.R., Pryor L.S., Sweeney W., Papay F.A., Zins J.E. (2010). Calcium phosphate cements in skull reconstruction: A meta-analysis. Plast. Reconstr. Surg..

[69] Xu H.H., Wang P., Wang L., Bao C., Chen Q., Weir M.D., Chow L.C., Zhao L., Zhou X., Reynolds M.A. (2017). Calcium phosphate cements for bone engineering and their biological properties. Bone Res..

[70] Amini A.R., Laurencin C.T., Nukavarapu S.P. (2012). Bone Tissue Engineering: Recent Advances and Challenges. Crit. Rev. Biomed. Eng..

[71] Du G.-Y., He S.-W., Sun C.-X., Mi L.-D. (2017). Bone Morphogenic Protein-2 (rhBMP2)-Loaded Silk Fibroin Scaffolds to Enhance the Osteoinductivity in Bone Tissue Engineering. Nanoscale Res. Lett..

[72] Chen J.-P., Chang G.-Y., Chen J.-K. (2008). Electrospun collagen/chitosan nanofibrous membrane as wound dressing. Colloids Surf. A Physicochem. Eng. Asp..

[73] Witte F., Kaese V., Haferkamp H., Switzer E., Meyer-Lindenberg A., Wirth C.J., Windhagen H. (2005). In vivo corrosion of four magnesium alloys and the associated bone response. Biomaterials.

[74] Witte F., Feyerabend F., Maier P., Fischer J., Störmer M., Blawert C., Dietzel W., Hort N. (2007). Biodegradable magnesium-hydroxyapatite metal matrix composites. Biomaterials.

[75] Huan Z.G., Leeflang M.A., Zhou J., Duszczyk J. (2011). ZK30-bioactive glass composites for orthopedic applications: A comparative study on fabrication method and characteristics. Mater. Sci. Eng. B Solid-State Mater. Adv. Technol..

[76] Huan Z., Zhou J., Duszczyk J. (2010). Magnesium-based composites with improved in vitro surface biocompatibility. J. Mater. Sci. Mater. Med..

[77] Rady A.A.M., Hamdy S.M., Abdel-Hamid M.A., Hegazy M.G.A., Fathy S.A., Mostafa A.A. (2020). The role of VEGF and BMP-2 in stimulation of bone healing with using hybrid bio-composite scaffolds coated implants in animal model. Bull. Natl. Res. Cent..

[78] Martín-del-Campo M., Rosales-Ibañez R., Rojo L. (2019). Biomaterials for Cleft Lip and Palate Regeneration. Int. J. Mol. Sci..

[79] Martín-Piedra M.A., Alaminos M., Fernández-Valadés-gámez R., España-López A., Liceras-Liceras E., Sánchez-Montesinos I., Martínez-Plaza A., Sánchez-Quevedo M.C., Fernández-Valadés R., Garzón I. (2017). Development of a multilayered palate substitute in rabbits: A histochemical Ex Vivo and In Vivo analysis. Histochem. Cell Biol..

[80] Janssen N.G., De Ruiter A.P., Van Hout W.M.M.T., Van Miegem V., Gawlitta D., De Groot F.B., Meijer G.J., Rosenberg A.J.W.P., Koole R. (2017). Microstructured β-Tricalcium phosphate putty versus autologous bone for repair of alveolar clefts in a goat model. Cleft Palate-Craniofacial J..

[81] Korn P., Ahlfeld T., Lahmeyer F., Kilian D., Sembdner P., Stelzer R., Pradel W., Franke A., Rauner M., Range U. (2020). 3D Printing of Bone Grafts for Cleft Alveolar Osteoplasty—In vivo Evaluation in a Preclinical Model. Front. Bioeng. Biotechnol..

[82] Amalraj J.C., Gangothri M., Babu H. (2017). Reconstruction of Drug-induced Cleft Palate Using Bone Marrow Mesenchymal Stem Cell in Rodents. Ann. Maxillofac. Surg..

[83] Caballero M., Morse J.C., Halevi A.E., Emodi O., Pharaon M.R., Wood J.S., van Aalst J.A. (2015). Juvenile Swine Surgical Alveolar Cleft Model to Test Novel Autologous Stem Cell Therapies. Tissue Eng. Part C Methods.

[84] Alkaabi S.A., Natsir Kalla D.S., Alsabri G.A., Fauzi A., Tajrin A., Müller W.E.G., Schröder H.C., Wang X.G., Forouzanfar T., Helder M.N. (2021). Polyphosphate (PolyP) for alveolar cleft repair: Study protocol for a pilot randomized controlled trial. Trials.

[85] Ahlfeld T., Lode A., Richter R.F., Pradel W., Franke A., Rauner M., Stadlinger B., Lauer G., Gelinsky M., Korn P. (2021). Toward Biofabrication of Resorbable Implants Consisting of a Calcium Phosphate Cement and Fibrin—A Characterization In Vitro and In Vivo. Int. J. Mol. Sci..

[86] Kamal M., Andersson L., Tolba R., Al-Asfour A., Bartella A.K., Gremse F., Rosenhain S., Hölzle F., Kessler P., Lethaus B. (2017). Bone regeneration using composite non-demineralized xenogenic dentin with beta-tricalcium phosphate in experimental alveolar cleft repair in a rabbit model. J. Transl. Med..

[87] Naudot M., Davrou J., Djebara A.-E., Barre A., Lavagen N., Lardière S., Azdad S.Z., Zabijak L., Lack S., Devauchelle B. (2020). Functional Validation of a New Alginate-based Hydrogel Scaffold Combined with Mesenchymal Stem Cells in a Rat Hard Palate Cleft Model. Plast. Reconstr. Surg. Glob. Open.

[88] Mostafa N.Z., Talwar R., Shahin M., Unsworth L.D., Major P.W., Doschak M.R. (2015). Cleft Palate Reconstruction Using Collagen and Nanofiber Scaffold Incorporating Bone Morphogenetic Protein in Rats. Tissue Eng. Part A.

[89] Li W., Fu Y., Jiang B., Lo A.Y., Ameer G.A., Barnett C., Wang B. (2019). Polymer-integrated amnion scaffold significantly improves cleft palate repair. Acta Biomater..

[90] Rizzo M.I., Tomao L., Tedesco S., Cajozzo M., Esposito M., De Stefanis C., Ferranti A.M., Mezzogori D., Palmieri A., Pozzato G. (2021). Engineered mucoperiosteal scaffold for cleft palate regeneration towards the non-immunogenic transplantation. Sci. Rep..

[91] Reddy G.S.P. (2016). Membrane Assisted Palatal Fistula Closure in a Cleft Palate Patient: A Novel Technique. J. Clin. Diagn. Res..

[92] Mossaad A., El Badry T., Abdelrahman M., Abd Elazeem A., Ghanem W., Hassan S., Adly N., Shawkat W. (2019). Alveolar Cleft Reconstruction Using Different Grafting Techniques. Open Access Maced. J. Med. Sci..

[93] Ahmed A., Gibson C., Ayliffe P. (2013). Technical note Use of polydioxanone sheet to repair palatal fistulas in patients with cleft palate. Br. J. Oral Maxillofac. Surg..

[94] Rodriguez I.A., Madurantakam P.A., McCool J.M., Sell S.A., Yang H., Moon P.C., Bowlin G.L. (2012). Mineralization potential of electrospun PDO-hydroxyapatite-fibrinogen blended scaffolds. Int. J. Biomater..

[95] Puwanun S., Delaine-Smith R.M., Colley H.E., Yates J.M., MacNeil S., Reilly G.C. (2018). A simple rocker-induced mechanical stimulus upregulates mineralization by human osteoprogenitor cells in fibrous scaffolds. J. Tissue Eng. Regen. Med..

[96] Sasayama S., Hara T., Tanaka T., Honda Y., Baba S. (2018). Osteogenesis of Multipotent Progenitor Cells using the Epigallocatechin Gallate-Modified Gelatin Sponge Scaffold in the Rat Congenital Cleft-Jaw Model. Int. J. Mol. Sci..

